# IGHV mutational status and BCR stereotypy in chronic lymphocytic leukemia: A Turkish cohort analysis

**DOI:** 10.1007/s00277-026-07096-9

**Published:** 2026-06-03

**Authors:** Seher Yüksel, Rahmi Şinasi Aygün, Merve Yüksel, Mehmet Berk Örüncü, Ezgi Dicle Serbes, Güldane Cengiz Seval, Önder Arslan, Muhit Özcan, Işınsu Kuzu

**Affiliations:** 1 Department of Pathology, School of Medicine, Ankara University, Ankara, Türkiye; 2 Biotechnology Institute, Ankara University, Ankara, Türkiye; 3 Department of Hematology, School of Medicine, Ankara University, Ankara, Türkiye; 4 Department of Oncology, School of Medicine, Ankara University, Ankara, Türkiye

**Keywords:** Chronic lymphocytic leukemia, IGHV mutational status, BCR stereotypy, Major stereotyped subsets, Real-world cohort

## Abstract

Immunoglobulin heavy chain variable region (IGHV) mutational status and B-cell receptor (BCR) stereotypy are established prognostic markers in chronic lymphocytic leukemia (CLL). However, immunogenetic data from Türkiye and surrounding regions are scarce. We evaluated IGHV mutational status, BCR stereotypy including nearest subset assignment, and their clinical and cytogenetic correlates in a real-world Turkish CLL cohort. We retrospectively analyzed 145 patients with CLL at a tertiary referral center in Türkiye. IGHV mutational status was determined by next-generation sequencing (98% germline homology cutoff). BCR stereotypy was assigned using ARResT/AssignSubsets. Cytogenetic abnormalities were assessed by fluorescence in situ hybridization (FISH). Time-to-first treatment (TTFT), progression-free survival (PFS), and overall survival (OS) were analyzed by Kaplan–Meier method, with multivariate Cox regression for TTFT. Unmutated IGHV was detected in 55.2% of patients, higher than Western reports but consistent with Mediterranean and Middle Eastern cohorts. Unmutated cases showed adverse cytogenetics—del(11q) and multiple concurrent FISH abnormalities—whereas del(13q) predominated in mutated cases. At a median follow-up of 43.0 months unmutated status strongly predicted a profoundly shorter median TTFT compared to mutated cases (17.0 vs. 70.0 months; p < 0.0001). Notably, while an unmutated IGHV status strongly predicted a significantly shorter TTFT, it did not translate into worse PFS or OS compared to mutated cases, reflecting the transformative real-world impact and rescue capacity of modern targeted therapies utilized upon disease progression. Major stereotyped subsets were present in 15.9% of patients; subset #2 was absent, while subset #1 was predominant (39.1%). Subsets #1 and #64B were overrepresented among relapsed cases. Nearest subset #77 showed rapid progression despite mutated IGHV. CLL in Türkiye demonstrates region-specific immunogenetic features while preserving established clinico-biological correlations. Integration of IGHV status, cytogenetics, and BCR stereotypy may improve risk stratification in underrepresented populations.

**Supplementary Information:**

The online version contains supplementary material available at 10.1007/s00277-026-07096-9.

## Introduction

Chronic lymphocytic leukemia (CLL) is an indolent B-cell malignancy accounting for approximately 30–40% of adult leukemias, yet its clinical course is remarkably heterogeneous [1]. While many patients remain stable for years without requiring therapy, others progress rapidly, highlighting the importance of reliable biological markers for prognostic stratification and treatment planning.

Contemporary risk assessment integrates the Rai and Binet staging systems with FISH-detected cytogenetic abnormalities—del(17p), del(11q), trisomy 12, and del(13q)—and recurrent gene mutations [2]. The prognostic significance of somatic hypermutation (SHM) within the immunoglobulin heavy chain variable region (IGHV) gene was established in 1999 [3, 4]; patients with unmutated IGHV (U-CLL; ≥ 98% germline homology) experience more aggressive disease and shorter TTFT, while mutated IGHV (M-CLL; < 98% homology) confers a more indolent trajectory [5, 6]—distinctions formalized in the CLL-IPI [7].

Immunogenetic studies have shown that approximately 40% of CLL patients express quasi-identical BCR immunoglobulins—a phenomenon termed BCR stereotypy [8, 9]. Subsets sharing VH CDR3 characteristics are classified as major (≥ 20 cases, reproducible clinical behavior) or minor [1, 8].

Despite extensive investigation in Western populations, real-world immunogenetic data from Türkiye remain scarce. We therefore analyzed a Turkish CLL cohort to characterize IGHV mutational status, IGHV gene repertoire, BCR stereotypy, and their clinical and cytogenetic correlates.

## Materials and methods

### Study design and patients

We enrolled consecutive patients diagnosed with CLL at our tertiary center (Ankara University School of Medicine) between 2019 and 2025, all of whom had undergone IGHV mutational status testing as part of routine clinical workup. Two exclusion criteria were applied to ensure the immunogenetic validity of our analysis: patients with exclusively unproductive IGHV rearrangements were excluded, as productive clones are required for BCR stereotyped subset assignment, and patients with a concurrent active secondary hematologic malignancy were also excluded. No additional clinical or demographic exclusion criteria were used. The final cohort consisted of 145 patients with productive rearrangements. The study was conducted in accordance with the Declaration of Helsinki and approved by the Ankara University Human Research Ethics Committee (Approval No: 2023000138-1 [2023/138]).

### Data collection and cytogenetics

Clinical and laboratory data were retrospectively obtained from electronic medical records. Baseline variables included age, sex, Rai and Binet stage at diagnosis, and absolute lymphocyte count. FISH analysis was performed on peripheral blood at the time of diagnosis using probes for del(13q), trisomy 12, del(11q), and del(17p); del(17p) was considered a high-risk cytogenetic abnormality.

The presence of multiple concurrent FISH abnormalities was operationalized based on our standard panel and defined as the synchronous detection of more than one cytogenetic lesion within the evaluated loci.

### IGHV mutational analysis

IGHV mutational status was determined in accordance with the recommendations of the European Research Initiative on CLL (ERIC). DNA was extracted from peripheral blood, bone marrow aspirates, or formalin-fixed paraffin-embedded (FFPE) tissue. Next-generation sequencing (NGS) was performed using the LymphoTrack^®^ Dx IGHV Somatic Hypermutation Assay Panel on the Illumina MiSeq platform. Sequences were analyzed using the IMGT database with a 98% homology cutoff. Only productive IGHV rearrangements were included; in patients with multiple productive clones, the dominant clone determined classification. Discordant cases (mutated and unmutated productive clones coexisting) were classified as U-CLL per ERIC recommendations. Dominant sequences were subsequently analyzed using the ARResT/AssignSubsets platform (http://arrest.tools/assignsubsets) for immunogenetic classification.

### BCR stereotypy analysis

Stereotyped BCR subset assignment was performed using the ARResT/AssignSubsets tool (http://arrest.tools/assignsubsets) based on IMGT-defined immunogenetic criteria, including IGHV gene clan usage, VH CDR3 length, amino acid identity, and physicochemical similarity. Cases fulfilling full assignment criteria were classified as major stereotyped subsets; those demonstrating immunogenetic similarity without meeting strict thresholds were designated nearest stereotyped subsets.

In cases presenting with multiple concurrent productive IGHV rearrangements (*n* = 15, 10.3%), subset assignment was operationalized using a dominant-clone approach rather than multiple assignments. Following international consensus recommendations, the rearrangement exhibiting the highest clonal frequency was selected as the sole representative clone for BCR stereotyped subset mapping. Minor productive sequences, even if displaying subclonal subset features, were excluded from the final analytical framework to prevent clinical and prognostic confounding.

### Statistical analysis

Categorical variables were compared using chi-square or Fisher’s exact tests; continuous variables were assessed using the Mann–Whitney U test or independent-samples t-test. Survival outcomes (TTFT, PFS, OS) were analyzed by Kaplan–Meier method with log-rank comparisons. Multivariate analysis of TTFT employed Cox proportional hazards regression incorporating age, sex, clinical stage, and high-risk cytogenetics. Analyses were performed using IBM SPSS Statistics version 26.0; survival curves were generated in Python 3.10 with Matplotlib. For univariate analyses, a variable-wise exclusion approach was utilized. For multivariable Cox regression models, missing data were handled using a complete-case analysis approach, whereby only patients with fully available profiles across all entered covariates were included in the final regression models.

## Results

### Patient characteristics

A total of 145 patients were included. Median age at diagnosis was 59.0 years (range, 30–93), with a male predominance (*n* = 88, 60.7%). IGHV status was unmutated in 80 patients (55.2%) and mutated in 65 patients (44.8%). Baseline characteristics stratified by IGHV status are summarized in Table 1. 

### Association of IGHV mutational status with clinical features

Mutated IGHV was associated with earlier clinical stage. In M-CLL, 32.3% presented at Rai stage 0 versus 16.2% in U-CLL, and Binet A was more frequent in M-CLL when compared with U-CLL (53.8% vs. 37.5%; *p* = 0.068). Risk stratification using the CLL-IPI revealed a significant association with IGHV mutational status (*p* < 0.001). High and very high-risk CLL-IPI scores were predominantly clustered within the unmutated group (72.0%), whereas these risk categories accounted for only 28.0% of patients with mutated IGHV. Lymphocyte count at diagnosis did not differ between groups (*p* = 0.573).

Cytogenetic data were available for 121 patients (83.4% of the cohort). Cytogenetic profiles showed distinct distributions by IGHV status. Del(13q) was significantly more frequent in M-CLL than U-CLL (66.7% vs. 37.5%; *p* = 0.004), while del(11q) predominated in U-CLL (27.0% vs. 8.0%; *p* = 0.014). Multiple cytogenetic abnormalities were more common in U-CLL (29.4% vs. 11.3%; *p* = 0.029). While trisomy 12 (27.4% vs. 14.9%) and del(17p) (16.7% vs. 9.4%) were numerically higher in U-CLL, these differences did not reach statistical significance (*p* = 0.162 and *p* = 0.290, respectively).

**Table 1:** Baseline demographic and clinical characteristics of the study cohort according to IGHV mutational status

Characteristics	Total cohort (*n* = 145)	IGHV mutated (*n* = 65)	IGHV unmutated (*n* = 80)	*p*-value
**Age**, years, median (range)	59.0 (30–93)	59.5 (33–86)	58.5 (30–93)	0.443
**Gender**				
Male, n (%)	88 (60.7%)	37 (56.9%)	51 (63.8%)	0.505
Female, n (%)	57 (39.3%)	28 (43.1%)	29 (36.2%)	
**Lymphocyte count** (cells/µL), median (range)	17,000 (1,730 − 420,000)	15,345 (1,730 − 420,000)	17,960 (2,330 − 294,710)	0.573
**Rai Stage**, n (%)				
Stage 0	34 (23.4%)	21 (32.3%)	13 (16.2%)	0.131
Stage I	55 (37.9%)	23 (35.4%)	32 (40.0%)	
Stage II	24 (16.6%)	7 (10.8%)	17 (21.2%)	
Stage III	19 (13.1%)	7 (10.8%)	12 (15.0%)	
Stage IV	8 (5.5%)	4 (6.2%)	4 (5.0%)	
Missing data	5 (3.4%)	3 (4.6%)	2 (2.5%)	
**Binet Stage**, n (%)				
Stage A	65 (44.8%)	35 (53.8%)	30 (37.5%)	0.068
Stage B	55 (37.9%)	18 (27.7%)	37 (46.2%)	
Stage C	20 (13.8%)	9 (13.8%)	11 (13.8%)	
Missing data	5 (3.4%)	3 (4.6%)	2 (2.5%)	
**Cytogenetics (FISH)**, n/eval (%)				
del(13q)	56/112 (50.0%)	32/48 (66.7%)	24/64 (37.5%)	0.004*
del(11q)	21/113 (18.6%)	4/50 (8.0%)	17/63 (27.0%)	0.014*
del(17p)	16/119 (13.4%)	5/53 (9.4%)	11/66 (16.7%)	0.290
Trisomy 12	24/109 (22.0%)	7/47 (14.9%)	17/62 (27.4%)	0.162
Multiple FISH abnormalities (≥ 2 aberrations)	26/121 (21.5%)	6/53 (11.3%)	20/68 (29.4%)	0.029*

Data are presented as number (percentage) for categorical variables and median (minimum–maximum) for continuous variables. Percentages were calculated based on the number of patients with available data in each group. *Statistically significant. Abbreviations: *CLL* Chronic lymphocytic leukemi, *FISH* Fluorescence in situ hybridization, *IGHV* Immunoglobulin heavy chain variable region

### IGHV gene repertoire

A total of 161 productive IGHV rearrangements were identified among 145 patients; 130 (89.7%) had a single productive rearrangement and 15 patients (10.3%) had multiple. The most frequently used IGHV genes were *IGHV4-34* (11.2%), *IGHV1-69* (8.1%), and *IGHV3-23* (6.8%). Gene usage differed by mutational status: *IGHV4-34* predominated in M-CLL (20.8%), and *IGHV1-69* in U-CLL (13.1%), as shown in Supplementary Table 1.

### Biclonal IGHV rearrangements and discordant IGHV mutational status

Among 15 patients with multiple productive rearrangements, 10 showed concordant mutational status and 5 (3.4%) were discordant harboring both mutated and unmutated clones (Supplementary Table 2). *IGHV4-34* was recurrent in discordant mutated clones. All five were classified as U-CLL per ERIC recommendations. Four of the five (80%) required systemic therapy with short TTFT (0, 8, 24, and 72 months), with clinical trajectories consistent with U-CLL.

## BCR stereotypy

### Major stereotyped BCR subsets

Major stereotyped BCR subsets were identified in 23 of 145 patients (15.9%) using ARResT/AssignSubsets. Subset #1 was most frequent (*n* = 9, 39.1%), followed by subsets #3 and #4 (each *n* = 3, 13.0%). Subsets #1, #3, #64B, #7H, #99, #31, and #8 were exclusive to U-CLL; subsets #4, #77, and #14 were confined to M-CLL (Table 2).

**Table 2:** Distribution of major stereotyped BCR subsets according to IGHV mutational status

Major subsets	All patients (*n* = 23), n (%)	Mutated IGHV, *n* (%)	Unmutated IGHV, *n* (%)
Subset #1	9 (39.1%)	0 (0%)	9 (100%)
Subset #3	3 (13.0%)	0 (0%)	3 (100%)
Subset #4	3 (13.0%)	3 (100%)	0 (0%)
Subset #64B	2 (8.7%)	0 (0%)	2 (100%)
Subset #7H	1 (4.3%)	0 (0%)	1 (100%)
Subset #77	1 (4.3%)	1 (100%)	0 (0%)
Subset #14	1 (4.3%)	1 (100%)	0 (0%)
Subset #99	1 (4.3%)	0 (0%)	1 (100%)
Subset #31	1 (4.3%)	0 (0%)	1 (100%)
Subset #8	1 (4.3%)	0 (0%)	1 (100%)
**Total**	**23 (100**%**)**	**5 (21.7**%**)**	**18 (78.3**%**)**

Percentages in the “Mutated IGHV” and “Unmutated IGHV” columns represent row-wise percentages

### Nearest stereotyped BCR subsets

Nearest stereotyped subsets were identified in 27 patients (28 total assignments). Nearest subsets #77 and #64B were most frequent (14.3% each). Unmutated IGHV predominated (67.9% of assignments): nearest subsets #64B, #6, #4, #8, and #202 were exclusive to U-CLL, while nearest subsets #77, #201, and #14 occurred only in M-CLL (Supplementary Table 3).

### Treatment modalities and relapse characteristics

Of 145 patients, 82 (56.6%) required first-line systemic therapy. Most (*n* = 53) received conventional chemoimmunotherapy, primarily fludarabine-based regimens (FCR), bendamustine plus rituximab (BR), chlorambucil-based combinations, or cyclophosphamide-containing protocols (R-CHOP, R-CVP). Upfront targeted therapy was administered in 26 patients, including covalent and non-covalent Bruton’s tyrosine kinase (BTK) inhibitors (ibrutinib, acalabrutinib, pirtobrutinib, nemtabrutinib) and the B-cell lymphoma 2 (BCL-2) inhibitor venetoclax, used as monotherapy or in fixed-duration combinations with anti-CD20 antibodies; detailed treatment distribution is provided in Supplementary Table 4.

Disease progression requiring second-line therapy occurred in 29 patients (35.4% of treated). Most had initially received fludarabine-based chemoimmunotherapy (FCR, *n* = 17), with smaller proportions receiving chlorambucil-based combinations (*n* = 6), BR (*n* = 3), or R-CVP (*n* = 2). At relapse, treatment shifted toward targeted approaches: ibrutinib-based regimens were most frequently used (*n* = 15), followed by BR (*n* = 7) and venetoclax-based combinations (*n* = 3).

The relapsed subgroup showed marked enrichment of adverse features: unmutated IGHV predominated (79.3%), del(11q) was present in 34.5%, and del(17p) in 17.2%. BCR stereotypy was identified in 13 of 29 relapsed patients. Subset #1 was the most frequent (*n* = 5), with relapse occurring in 5 of the 6 treated subset #1 patients in the overall cohort. Subsets #64B (*n* = 2) and #8 (*n* = 1) were also identified among relapsed cases. Among nearest subsets, nearest subset #64B (*n* = 3) and nearest subset #4 (*n* = 2) were represented, collectively suggesting that subsets #1 and #64B may be associated with heightened susceptibility to early treatment failure.

### Survival analyses

**Fig. 1:**
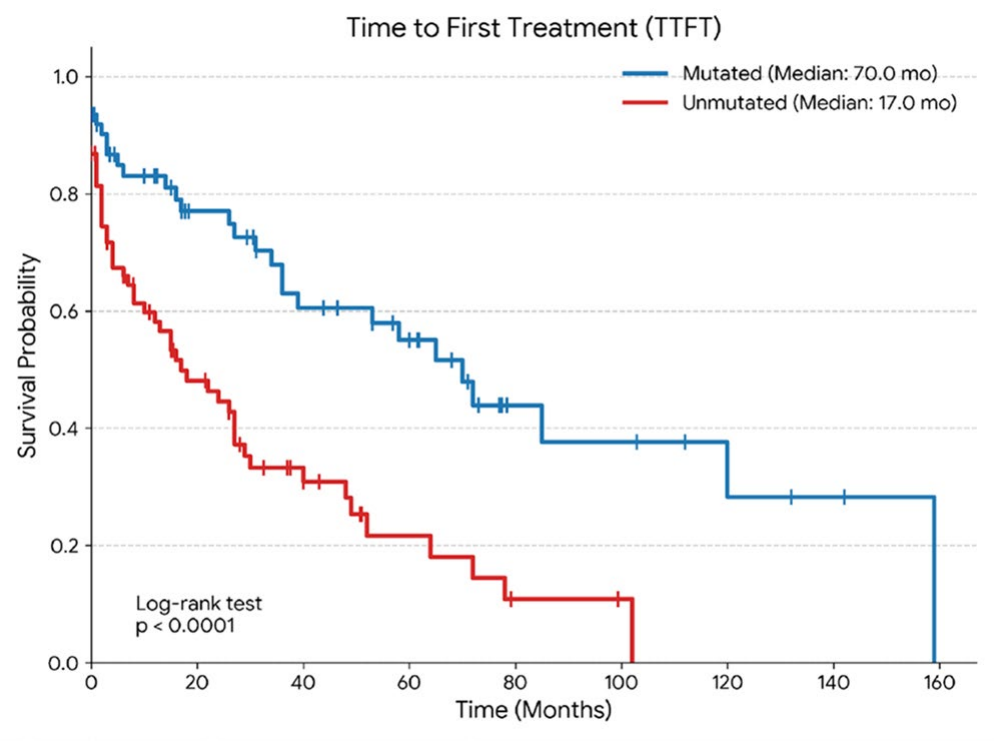
Kaplan-Meier curves for time to treatment (TTFT) stratified by IGHV mutational status. The curve evaluates the entire cohort of 145 patients (U-CLL: n=80; M-CLL: n=65) at baseline (Time 0). The overall median follow-up of the cohort was 43.0 months (range: 0–191 months). Patients who did not require treatment during their follow-up period were dynamically incorporated into the model as censored observations based on their total follow-up durations. U-CLL patients exhibited a significantly shorter median TTFT compared to M-CLL patients (17.0 months vs. 70.0 months; log-rank p < 0.0001). Note: Subsequent multivariable Cox regression models automatically restricted execution to n=89 complete cases due to concurrent clinical covariates

Median follow-up was 43.0 months (range: 0–191). U-CLL patients had significantly shorter median TTFT than M-CLL (17.0 vs. 70.0 months; log-rank *p* < 0.0001; Fig. 1). Multivariate Cox regression confirmed unmutated IGHV as an independent predictor among the evaluated complete cases (*n* = 89): Model 1 (Binet) HR 2.39 (95% CI 1.38–4.14; *p* = 0.0018), Model 2 (Rai) HR 2.28 (95% CI 1.31–3.95; *p* = 0.0034). Advanced clinical stage and del(11q) were also independent predictors in both models (*p* < 0.05).

**Fig. 2:**
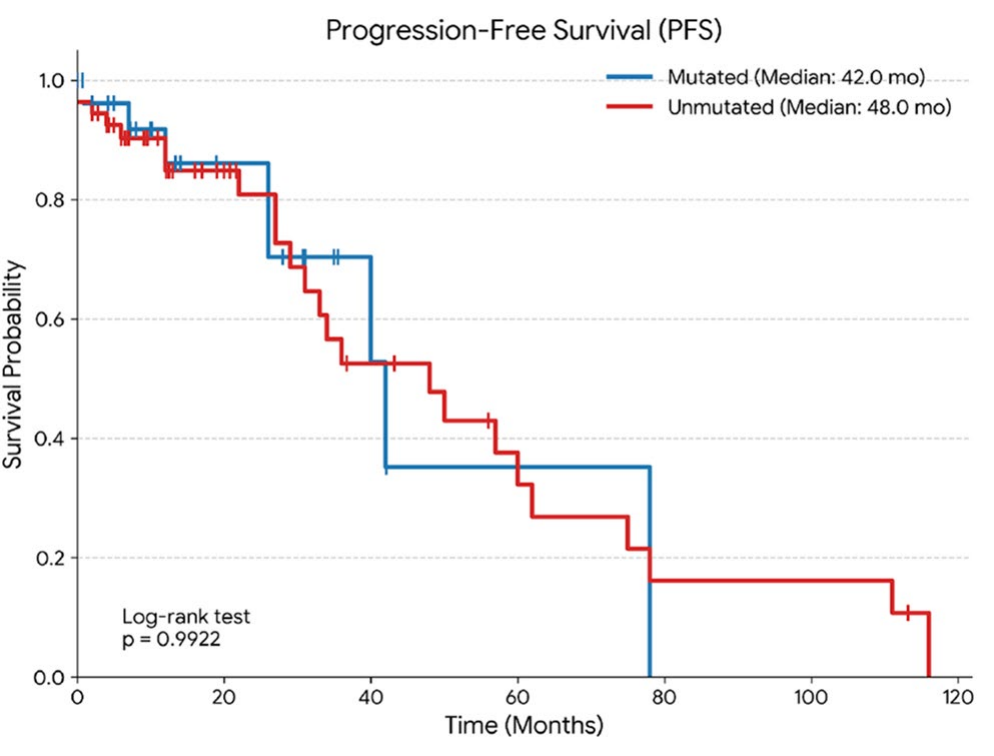
Kaplan-Meier curves for progression-free survival (PFS) in treated patients stratified by IGHV mutational status. The post-treatment progression dynamics evaluate the sub-cohort of treated patients with evaluable records (*n* = 45; U-CLL: *n* = 28; M-CLL: *n* = 17 at Time 0). No statistically significant difference in PFS was observed between U-CLL and M-CLL groups (median 48.0 vs. 42.0 months; log-rank *p* = 0.9922)

**Fig. 3:**
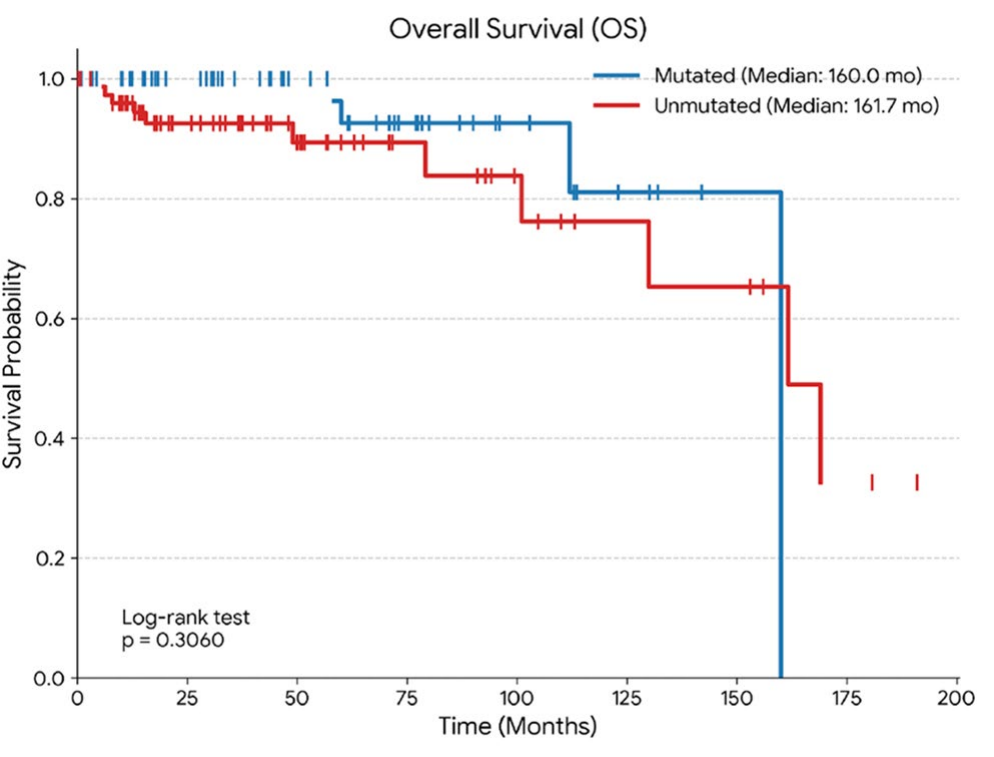
Kaplan-Meier curves for overall survival (OS) by IGHV mutational status. The survival infrastructure examines long-term clinical survival profiles across a total of 139 evaluable cases (U-CLL: *n* = 76 at risk; M-CLL: *n* = 63 at risk at baseline), with 6 cases excluded from the total cohort due to missing or unavailable follow-up parameters. Overall survival demonstrated equivalent outcomes between the two immunogenetic categories (log-rank *p* = 0.3060)

Notably, despite this profoundly shorter TTFT in the unmutated category, long-term post-treatment outcomes did not significantly differ between the groups. PFS showed no significant difference between U-CLL and M-CLL (median 48.0 vs. 42.0 months; *p* = 0.9922; Fig. 2). OS was likewise similar between groups (log-rank *p* = 0.3060; Fig. 3), demonstrating a clear survival paradox that is highly consistent with the real-world efficacy of modern targeted therapies in completely mitigating and attenuating the historically adverse prognostic impact of unmutated IGHV upon disease progression.

Among relapsed patients (*n* = 29), 79.3% harbored unmutated IGHV, del(11q) was present in 34.5%, and del(17p) in 17.2%. Subsets #1 and #64B were overrepresented, with relapse in 5 of 6 treated subset #1 patients, suggesting BCR stereotypy as a marker of early treatment failure.

## Discussion

This study characterizes IGHV mutational status, clonal architecture, and BCR stereotypy in a Turkish CLL cohort. The proportion of U-CLL (55.2%) exceeded the approximately 40% reported in Western populations yet closely resembled frequencies in Mediterranean and Middle Eastern series [9, 10], supporting the concept of population-specific immunogenetic variability in CLL.

The median age at diagnosis (59 years) was lower than the 70–72 years reported in Western studies [7, 11, 12], mirroring figures from African and East Asian populations [13, 14], and may partly reflect tertiary referral dynamics that concentrate younger, biologically complex patients.

The IGHV gene repertoire mirrored Western and Mediterranean data—*IGHV4-34* predominating in M-CLL, *IGHV1-69* enriched in U-CLL—consistent with antigen-driven selection [6, 9, 15–17]. Oligoclonality (10.3%) fell within expected ranges [18]. Discordant mutational profiles were observed in one-third of oligoclonal cases, with U-CLL-like trajectories, suggesting that minor unmutated subclones may carry independent prognostic relevance.

Among the most distinctive findings was the absence of stereotyped subset #2—prevalent in large Western cohorts [1, 6, 9, 19]—whereas subset #1 was the most frequent major subset (6.2%). Consistent with prior reports [6, 9, 16, 20–22], subset #1 patients uniformly harbored unmutated IGHV, presented at a younger age (median 56 years), and frequently exhibited del(17p) and trisomy 12. Richter transformation was observed in two patients; the case belonging to subset #8—carrying both del(17p) and trisomy 12 and dying 13 months after diagnosis—aligns with the aggressive biology and transformation risk associated with this subset [1, 6, 23–25].

The absence of subset #2 warrants consideration. In large European series this subset accounts for 2–5% of CLL cases and carries adverse prognosis linked to unmutated IGHV and del(11q) [1, 9, 19]. Two explanations may apply. First, stochastic variation cannot be excluded: with 145 patients, expected subset #2 cases would number two to seven, and sampling variability alone may explain non-detection. Second, population-level differences in HLA haplotype composition and microbial antigen exposure—both implicated in shaping the B-cell repertoire available for antigen selection—may reduce the frequency of this configuration [6, 16]. Geographic variation in subset #2 prevalence has been noted in prior studies, with some Mediterranean and East Asian cohorts similarly reporting low or absent representation [25, 26]. Confirmation requires larger multicenter Turkish series.

Subset #3 cases uniformly displayed unmutated IGHV and del(11q), consistent with their established adverse phenotype [2, 27]. Subset #4, by contrast, followed a reproducibly indolent course: all three cases harbored mutated IGHV with isolated del(13q), and none required therapy during follow-up [1, 24, 28].

Nearest stereotyped subsets showed clinically meaningful heterogeneity. Nearest subset #64B was invariably associated with aggressive disease and unmutated IGHV, while nearest subset #77 demonstrated rapid progression despite exclusive occurrence in M-CLL patients (median TTFT 1.5 months)—suggesting intrinsic BCR-driven aggressiveness that may override the protective effect of somatic hypermutation. Nearest subset #4 cases similarly diverged from the canonical indolent phenotype, primarily due to unmutated IGHV, underscoring that immunogenetic proximity does not guarantee equivalent clinical behavior.

Cytogenetic findings were concordant with international data: del(11q) and genomic complexity enriched in U-CLL, del(13q) predominating in M-CLL (11, 15, 28–30). Replication of these associations in a Turkish cohort confirms that the relationship between IGHV status and cytogenetic risk transcends geographic boundaries. Within nearest subset matches, cytogenetic profiles were less predictable, with occasional discordance from expected lesion patterns, illustrating that immunogenetic similarity does not ensure cytogenetic or clinical homogeneity.

Treatment failure concentrated among U-CLL patients with adverse cytogenetics; nearly 80% of relapsed cases harbored unmutated IGHV, reflecting the limited durability of chemoimmunotherapy in this population. At progression, treatment shifted toward targeted approaches—primarily BTK inhibitors and venetoclax-based regimens—and the absence of OS differences between groups is consistent with their established capacity to mitigate the survival disadvantage historically associated with unmutated IGHV [6, 20, 29, 30].

The lack of significant differences in PFS and OS across immunogenetic categories should be interpreted with caution, given the considerable treatment heterogeneity within our cohort. Patients received a wide range of frontline and subsequent regimens, from conventional chemoimmunotherapy to BTK inhibitors and venetoclax-based combinations, each with meaningfully different efficacy profiles. This heterogeneity in treatment exposure may have diluted or obscured subtle IGHV-dependent prognostic effects on post-treatment outcomes. That said, these findings are consistent with growing real-world evidence suggesting that modern targeted therapies largely overcome the adverse prognostic impact traditionally associated with unmutated IGHV status [6, 20, 29, 30].

In practical terms, these immunogenetic insights offer clear clinical guidance for hematologists in Türkiye and the wider region. In the era of targeted agents—where BTK inhibitors and BCL-2 antagonists have largely overcome historical survival disadvantages—evaluating IGHV status and BCR stereotypy should directly guide front-line therapeutic choices rather than serving as a mere prognostic exercise. For regional clinicians, identifying an unmutated IGHV status or an aggressive signature (such as subset #1 or #64B) mandates against traditional chemoimmunotherapy due to short-lived remissions and clonal evolution risks. Conversely, recognizing high-risk alignments like the nearest subset #77 within an otherwise indolent mutated profile alerts the clinician to maintain heightened vigilance for early progression. Consequently, routine immunogenetic screening empowers clinicians to bypass conventional chemotherapy in favor of upfront targeted protocols, optimizing personalized survival pathways.

Several limitations deserve acknowledgment. First, the retrospective, single-center design inherently introduces selection bias. As a major tertiary referral center in Türkiye, our institution tends to attract younger patients with biologically complex and advanced disease, which is reflected in the relatively young median age of our cohort (59 years). Patients were included on the basis of having available and productive IGHV mutational analysis, with the only clinical exclusion being the presence of a concurrent active secondary hematologic malignancy. As such, this cohort reflects the real-world molecular and clinical experience of a tertiary center rather than a community-based population. Additionally, FISH data were unavailable for approximately 20% of the patients, although this missingness was found to be largely at random and did not skew the overall survival analyses. Furthermore, while our sample size is reasonable for global cohort correlations, it remains limited for intra-subset level analyses; the small numbers within individual stereotyped subsets and nearest-subset groups mean that subset-specific clinical signals (e.g., subset #1, subset #64B, and nearest subset #77) should be appropriately viewed as hypothesis-generating rather than definitive markers. Despite these limitations, our study provides valuable, comprehensive real-world data from an underrepresented geographic region.

In summary, CLL in Türkiye exhibits immunogenetic features that are regionally distinct yet biologically coherent with established clinico-molecular correlations. Integrating IGHV mutational status, BCR stereotypy, clonal architecture, and cytogenetic profiling may support more refined risk stratification. Multicenter prospective studies are needed to define how immunogenetic risk translates into outcomes in the era of targeted therapies.

## Supplementary Information

Below is the link to the electronic supplementary material.

Supplementary Material 1

## Data Availability

No datasets were generated or analysed during the current study.
