# Supplementary material for: IGHV mutational status and BCR stereotypy in chronic lymphocytic leukemia: A Turkish cohort analysis
Source: Ann Hematol. 2026 Jun 3;105(8):361. doi: 10.1007/s00277-026-07096-9 (PMC13447546; doi:10.1007/s00277-026-07096-9)
Supplement: Supplementary file 1 [file 277_2026_7096_MOESM1_ESM.docx]

**Supplementary Tables**

**Supplementary Table 1. Frequency of IGHV gene usage according to mutational status**

| **IGHV Gene** | **Total (n=161)** | **Mutated IGHV (n=77)** | **Unmutated IGHV (n=84)** |
| --- | --- | --- | --- |
| ***IGHV4-34*** | 18 (11.2%) | 16 (20.8%) | 2 (2.4%) |
| ***IGHV1-69*** | 13 (8.1%) | 2 (2.6%) | 11 (13.1%) |
| ***IGHV3-23*** | 11 (6.8%) | 8 (10.4%) | 3 (3.6%) |
| ***IGHV4-59*** | 10 (6.2%) | 5 (6.5%) | 5 (6.0%) |
| ***IGHV3-21*** | 9 (5.6%) | 4 (5.2%) | 5 (6.0%) |
| ***IGHV4-39*** | 8 (5.0%) | 3 (3.9%) | 5 (6.0%) |
| ***IGHV1-2*** | 8 (5.0%) | 1 (1.3%) | 7 (8.3%) |
| ***IGHV3-7*** | 8 (5.0%) | 5 (6.5%) | 3 (3.6%) |
| ***IGHV3-48*** | 6 (3.7%) | 1 (1.3%) | 5 (6.0%) |
| ***IGHV3-30*** | 6 (3.7%) | 5 (6.5%) | 1 (1.2%) |

***Abbreviations:*** *IGHV, immunoglobulin heavy chain variable region* ***Data:*** *Values are presented as number of sequences (frequency). The total number of rearrangements (n=161) exceeds the number of patients (n=145) due to the detection of multiple productive clones in 15 patients*.

**Supplementary Table 2. Molecular characteristics of patients with discordant IGHV status**

| **Patient ID** | **V-gene Allele** | **Assigned Subset** | **Mutational Status** | **Clone size (% of total reads)** |
| --- | --- | --- | --- | --- |
| 16 | *IGHV3-9*01* | Unassigned | Unmutated | 47.12% |
|  | *IGHV4-34*01* | Unassigned | Mutated | 2.99% |
| 71 | *IGHV1-69*01* | Unassigned | Unmutated | 33.56% |
|  | *IGHV4-34*01* | Unassigned | Mutated | 4.03% |
| 73 | *IGHV5-51*01* | Unassigned | Mutated | 5.35% |
|  | *IGHV3-7*01* | Unassigned | Unmutated | 5.19% |
| 119 | *IGHV2-5*02* | Subset #14 | Mutated | 13.31% |
|  | *IGHV4-59*01* | Unassigned | Unmutated | 6.23% |
|  | *IGHV3-30*01* | Unassigned | Mutated | 4.77% |
| 136 | *IGHV3-66*01* | Subset #64B | Unmutated | 39.02% |
|  | *IGHV3-23*01* | Unassigned | Mutated | 4.24% |

***Abbreviations:*** *IGHV, immunoglobulin heavy chain variable region. Note: Mutated status is defined as <98% identity to the germline sequence.*

**Supplementary Table 3. Distribution of nearest stereotyped BCR subset rearrangements according to IGHV mutational status**

| **Nearest subsets** | **All rearrangements (n=28) n (%)** | **Mutated IGHV, n (%)** | **Unmutated IGHV, n (%)** |
| --- | --- | --- | --- |
| Nearest subset #64B | 4 (14.3%) | 0 (0%) | 4 (100%) |
| Nearest subset #77 | 4 (14.3%) | 4 (100%) | 0 (0%) |
| Nearest subset #6 | 3 (10.7%) | 0 (0%) | 3 (100%) |
| Nearest subset #201 | 3 (10.7%) | 3 (100%) | 0 (0%) |
| Nearest subset #4 | 2 (7.1%) | 0 (0%) | 2 (100%) |
| Nearest subset #8 | 2 (7.1%) | 0 (0%) | 2 (100%) |
| Nearest subset #14 | 2 (7.1%) | 2 (100%) | 0 (0%) |
| Nearest subset #202 | 2 (7.1%) | 0 (0%) | 2 (100%) |
| Nearest subset #1 | 1 (4.3%) | 0 (0%) | 1 (100%) |
| Nearest subset #3 | 1 (4.3%) | 0 (0%) | 1 (100%) |
| Nearest subset #7H | 1 (4.3%) | 0 (0%) | 1 (100%) |
| Nearest subset #12 | 1 (4.3%) | 0 (0%) | 1 (100%) |
| Nearest subset #28A | 1 (4.3%) | 0 (0%) | 1 (100%) |
| Nearest subset #31 | 1 (4.3%) | 0 (0%) | 1 (100%) |
| **Total** | **28 (100%)** | **9 (32.1%)** | **19 (67.9%)** |

*Percentages in the “Mutated IGHV” and “Unmutated IGHV” columns are calculated row-wise and represent the distribution of IGHV mutational status within each nearest stereotyped subset. Percentages in the “All rearrangements” column are calculated based on the total number of nearest subset rearrangements (n = 28). One patient contributed two nearest subset assignments.*

**Supplementary Table 4. First- and second-line treatment distribution according to IGHV mutational status**

| **Treatment** | **Patients who received first-line therapy (n=82)** | |
| --- | --- | --- |
|  | **IGHV mutated (n=29, 35.4%)** | **IGHV unmutated (n=53, 64.6%)** |
| **Chemoimmunotherapy** | 19 (65.5%) | 35 (66.0%) |
| FCR | 10 (34.5%) | 19 (35.8%) |
| FC | 0 (0%) | 2 (3.8%) |
| F | 1 (3.4%) | 0 (0%) |
| BR | 4 (13.8%) | 7 (13.2%) |
| Rituximab + Chlorambucil | 3 (10.3%) | 2 (3.8%) |
| Chlorambucil + Prednisolone | 1 (3.4%) | 1 (1.9%) |
| Chlorambucil | 0 (0%) | 1 (1.9%) |
| R-CHOP | 0 (0%) | 1 (1.9%) |
| R-CVP | 0 (0%) | 2 (3.8%) |
| **Targeted therapy** | 10 (34.5%) | 16 (30.2%) |
| Ibrutinib | 3 (10.4%) | 8 (15%) |
| Ibrutinib + Venetoclax | 1 (3.4%) | 1 (1.9%) |
| Obinutuzumab + Venetoclax | 2 (6.9%) | 2 (3.8%) |
| Obinutuzumab + Chlorambucil | 0 (0%) | 2 (3.8%) |
| Acalabrutinib | 1 (3.4%) | 1 (1.9%) |
| Acalabrutinib + Venetoclax | 1 (3.4%) | 0 (0%) |
| Rituximab + Venetoclax | 1 (3.4%) | 0 (0%) |
| Venetoclax | 0 (0%) | 1 (1.9%) |
| Pirtobrutinib | 0 (0%) | 1 (1.9%) |
| Nemtabrutinib | 1 (3.4%) | 0 (0%) |
| **Other** | 0 (0%) | 2 (3.8%) |
| Allogeneic SCT | 0 (0%) | 1 (1.9%) |
| **Treatment** | **Patients who received second-line therapy (n=29)** | |
|  | **IGHV mutated (n=6, 20.7%)** | **IGHV unmutated (n=23, 79.3%)** |
| Ibrutinib | 3 (50.0%) | 11 (47.8%) |
| BR | 3 (50.0%) | 4 (17.4%) |
| Ibrutinib + Venetoclax | 0 (0%) | 1 (4.3%) |
| Rituximab + Venetoclax | 0 (0%) | 2 (8.7%) |
| R-CHOP | 0 (0%) | 1 (4.3%) |
| R-CHOP + Ibrutinib | 0 (0%) | 1 (4.3%) |
| Chlorambucil + Prednisolone | 0 (0%) | 1 (4.3%) |
| Rituximab | 0 (0%) | 1 (4.3%) |
| Idelalisib + Rituximab | 0 (0%) | 1 (4.3%) |

*Abbreviations: FCR, fludarabine-cyclophosphamide-rituximab; FC, fludarabine-cyclophosphamide; BR, bendamustine-rituximab; R-CHOP, rituximab-cyclophosphamide-doxorubicin-vincristine-prednisone; R-CVP, rituximab-cyclophosphamide-vincristine-prednisone; SCT, stem cell transplantation. Percentages for first-line therapy are calculated out of all treated patients (n=82). Percentages for chemoimmunotherapy and targeted therapy subgroups are row-level. Second-line percentages are based on relapsed patients (n=29).*
